# Proteomic Remodeling in Fontan Circulation Associated with Fibrosis and Time-Dependent Immune-Metabolic Alteration †

**DOI:** 10.3390/ijms27167461

**Published:** 2026-08-20

**Authors:** Isabel Mihajlovic, David Renaud, Alexander Kirchmair, Fatima Ageed, Bettina Sarg, Klaus Faserl, Dietmar Rieder, Alexander Blaha, Nele Ströbel, Christian Lechner, Kai Thorsten Laser, Miriam Michel

**Affiliations:** 1 Department of Child and Adolescent Health, Division of Pediatrics III—Cardiology, Pulmonology, Allergology and Cystic Fibrosis, Medical University of Innsbruck, 6020 Innsbruck, Austria; isabel.mihajlovic@student.i-med.ac.at (I.M.); david.renaud.chd@gmail.com (D.R.); fatimaageed88@gmail.com (F.A.); alexander.blaha@student.i-med.ac.at (A.B.); nele.stroebel@student.i-med.ac.at (N.S.); 2 Biocenter, Institute of Bioinformatics, Medical University of Innsbruck, 6020 Innsbruck, Austria; alexander.kirchmair@i-med.ac.at (A.K.); dietmar.rieder@i-med.ac.at (D.R.); 3 Biocenter, Protein Core Facility, Medical University of Innsbruck, 6020 Innsbruck, Austria; bettina.sarg@i-med.ac.at (B.S.); klaus.faserl@i-med.ac.at (K.F.); 4 Department of Pediatrics, Division of Pediatric I—Neurology, Medical University of Innsbruck, 6020 Innsbruck, Austria; ch.lechner@i-med.ac.at; 5 Department for Children and Adults with Congenital Heart Disease (CHD), DOC Dortmund, Ruhr University Bochum, 44137 Dortmund, Germany; laser@kinderkardiologen.nrw

**Keywords:** endothelial dysfunction, extracellular matrix, fibrosis, Fontan circulation, immune activation, metabolic remodeling, proteomics, redox signaling, TGF-β

## Abstract

The Fontan circulation is associated with progressive multisystem complications, yet its underlying molecular mechanisms remain incompletely understood. We aimed to characterize systemic proteomic alterations and identify biological pathways associated with Fontan physiology, long-term adaptation, and clinically unfavorable phenotypes. Peripheral vein serum proteomics was performed using mass spectrometry in 48 Fontan patients and matched controls. Differential protein expression and pathway enrichment analyses were applied across the overall cohort and clinically defined subgroups according to Fontan duration and composite clinical risk phenotyping. More than one third of quantified proteins were differentially expressed, demonstrating extensive systemic proteomic remodeling. Extracellular matrix remodeling emerged as the dominant signature, characterized by fibrosis-associated proteins and enrichment of TGF-β-related profibrotic pathways. Prolonged Fontan duration and clinically unfavorable status were not associated with substantially amplified extracellular matrix signatures. Instead, long-term Fontan patients demonstrated increasing immune, complement, and endothelial activation, whereas high-risk patients showed metabolic and redox-related alterations. These findings indicate that extracellular matrix remodeling and profibrotic signaling are dominant features of the Fontan circulation, while increasing Fontan duration and clinical deterioration are associated with immune activation, endothelial stress, and metabolic dysregulation with oxidative stress.

## 1. Introduction

The long-term physiology following the Fontan procedure, the final step of the staged palliation for patients with complex single-ventricle congenital heart defects, represents a unique circulatory state characterized by elevated central venous pressure, lack of pulsatile pulmonary artery blood flow, limited cardiac output, and progressive multiorgan involvement. Despite major improvements in short-term outcomes [1], many individuals with a Fontan circulation develop complications over time [2], including enteric protein loss, cardiac or hepatic fibrosis, and metabolic dysregulation with abnormal glucose and lipid metabolism already in childhood [3]. Accordingly, initial molecular insights suggest that widespread molecular remodeling accompanies Fontan. Particularly recent proteomic investigations in the Fontan circulation have progressively expanded the functional landscape of the disease.

Initial large-scale plasma proteome profiling by Kelly et al. identified prominent alterations in pathways related to inflammation, angiogenesis, and vascular remodeling, thereby establishing a primarily inflammatory and vasculopathic disease paradigm [4], with hints towards metabolic involvement, particularly via lipid metabolism. Subsequently, in a proteomics platform comparison, Assi et al. demonstrated that many of these signals are sensitive to the underlying affinity-based measurement technology, particularly for cytokines and growth factors, highlighting the importance of platform-dependent bias and the need for cautious biological interpretation [5]. More recently, our group’s untargeted serum proteomics on a similarly heterogeneous group of Fontan patients broadened this perspective, revealing additional layers of dysregulation involving extracellular matrix (ECM) remodeling, cytoskeletal organization, platelet biology, and metabolic/redox pathways [6]. These findings suggest that the Fontan circulation extends beyond vascular inflammation towards a complex, multisystem disorder involving structural, cellular, and metabolic components, potentially particularly relevant when it comes to subgroup phenotyping. While our prior analyses provided important insights into network-defined protein clusters, such approaches may impose structural constraints on high-dimensional data. In particular, discretizing the proteome into predefined interaction networks can miss disease-specific biological variation and cross-system relationships. These are limitations that may become especially relevant in diseases such as the Fontan circulation, where biology is still not entirely understood and where no single physiological comparator exists. Inspired by work in other complex diseases, where unbiased analyses have helped identify previously unrecognized disease subtypes and biological mechanisms [7], we therefore applied a complementary, hypothesis-free analytical framework that leverages the full, continuous structure of the proteome without reliance on predefined interaction networks. In this cross-sectional study, we aimed to further refine the functional landscape of the Fontan circulation and to identify proteomic signatures associated with time since Fontan operation and with clinically defined subgroups, treating these findings as associations without inferring temporal progression or causality.

## 2. Results

Patient characteristics are summarized in Table 1. The Fontan cohort with 48 individuals had a mean age of 18 years, with 44% female, which was comparable to the age- and sex-matched control population. The mean time elapsed since Fontan completion was 14.9 years. Approximately half of the patients had a morphologic right ventricle. Fontan-related risk, as assessed using the 12-item composite score, was favorable (≤3) in the majority (77%) of patients. Most patients were receiving anticoagulation with vitamin K antagonists (90%), and a minority were on antiplatelet therapy (8%), with one patient receiving neither. A more comprehensive overview of the participant characteristics is provided in Supplementary Table S1. All participants were included in the final proteomic analysis, and no samples were excluded after processing. Because some clinical parameters were not available for all individuals, the number of observations varied between variables.

### 2.1. Multivariate Statistics

**Figure 1:**
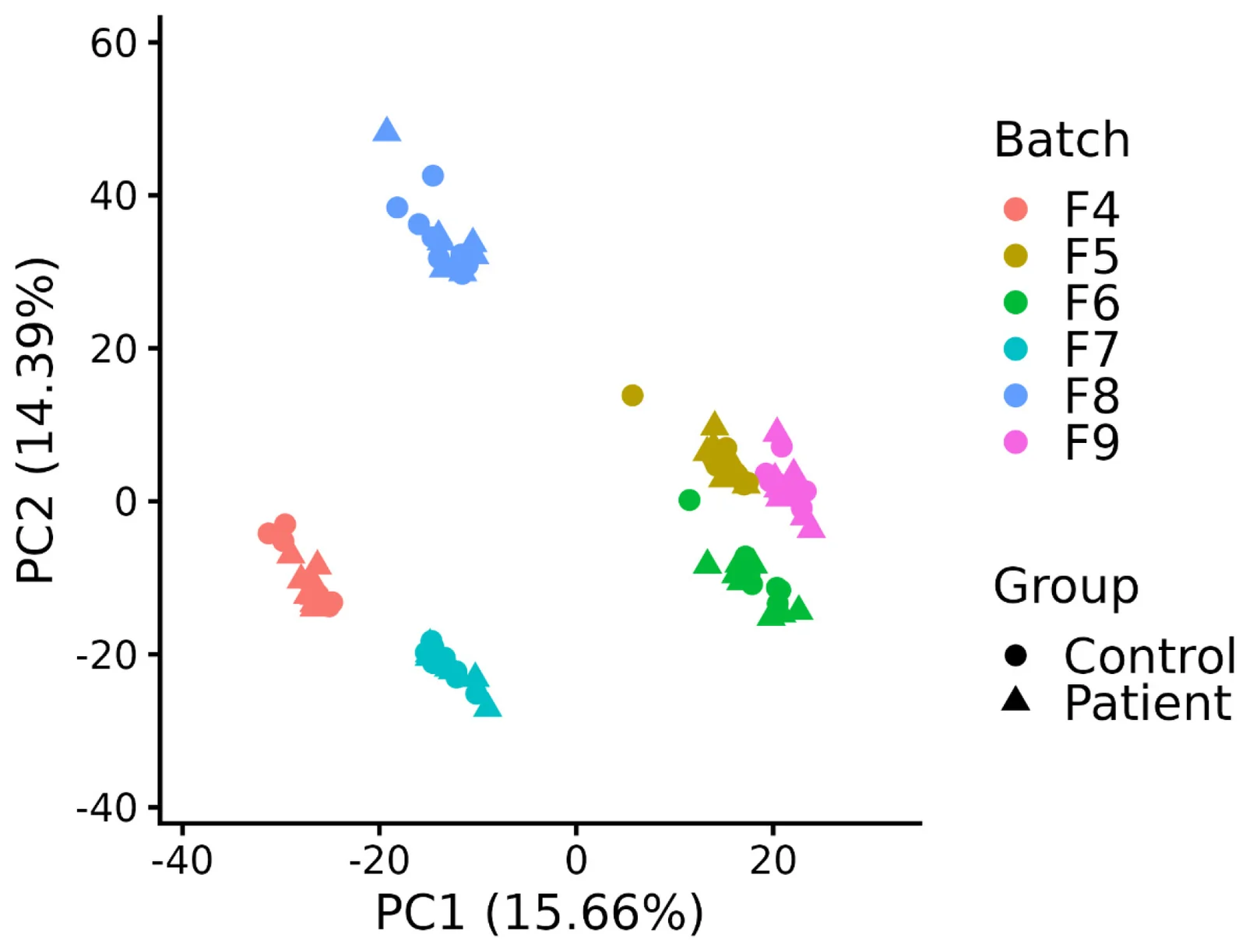
Principal component analysis (PCA) of log_2_-transformed protein abundances after data preprocessing (n = 1070 analytes). PC1, principal component 1; PC2, principal component 2. Patients are shown as triangles and controls as circles. The percentage of total variance explained by each principal component is indicated in parentheses. The samples were acquired in six TMT batches (IDs F4–F9), the colors denote the respective TMT batch. The non-consecutive IDs reflect internal numbering in Proteome Discoverer and do not imply missing batches in this dataset. Batch-related variation was accounted for during data processing. In this cohort, recruitment site is identical to disease status (patient or control); therefore, a separate ‘site effect’ is not estimable.

The principal component analysis (PCA) demonstrated marked differences between the batches (Figure 1), indicating increased proteomic variance within batches (each batch comprises samples from both patients and controls).

### 2.2. Serum Proteome of Patients vs. Controls

**Figure 2:**
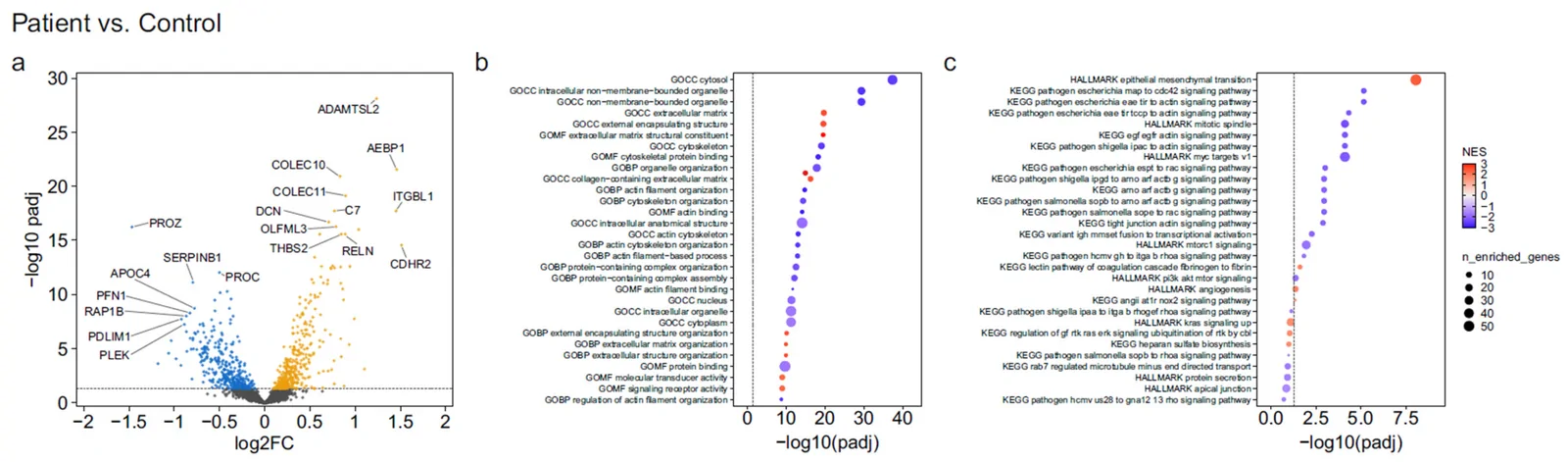
Serum proteome of Fontan patients compared with controls. (**a**) Volcano plot of differential protein abundance between patients and controls. Positive log_2_ fold change (log_2_FC) values indicate higher protein abundance, whereas negative values indicate lower abundance in patients (compared to controls). The y-axis represents statistical significance (−log_10_ adjusted *p*-value), and the dashed horizontal line indicates the significance threshold (adjusted *p*-value = 0.05). (**b**) Gene set enrichment analysis (GSEA) of Gene Ontology (GO) categories. Red dots indicate positively enriched gene sets (normalized enrichment score, NES > 0), whereas blue dots indicate negatively enriched gene sets (NES < 0). The x-axis displays the statistical significance of enrichment (−log_10_ adjusted *p*-value). (GOBP = GO biological process, GOCC = GO cellular component, GOMF = GO molecular function). (**c**) GSEA of MSigDB (Molecular Signatures Database) Hallmark and KEGG (Kyoto Encyclopedia of Genes and Genomes) pathways. Red dots indicate positively enriched pathways (NES > 0), whereas blue dots indicate negatively enriched pathways (NES < 0). The x-axis displays the statistical significance of enrichment (−log_10_ adjusted *p*-value). Abbreviations according to UniProt [8].

Out of the 2288 proteins quantified (of which 1070 were partially imputed), 808 were significantly altered in patients compared with controls (q < 0.05 for differential expression). A total of 372 proteins were significantly upregulated, while 436 were significantly downregulated compared to controls. These results are visualized in Figure 2 and reported in detail in Supplementary Tables S2–S4, Sheet S2, in each of those tables.

Using preranked GSEA with the measured proteome as the background, the strongest upregulated proteins were ECM-associated, including ADAMTS-Like Protein 2 (ADAMTSL2, ~2.3-fold), AE Binding Protein 1 (AEBP1, ~3-fold), Thrombospondin-2 (THBS2), Integrin Beta-Like Protein 1 (ITGBL1), Decorin (DCN), and Reelin (RELN, ~1.6–2.8-fold increases, all adj. *p*-values < 10^−15^) (Figure 2). Other notable elevations included Collectin-10 (COLEC10), Collectin-11 (COLEC11), and ADAM Metallopeptidase with Thrombospondin Type 1 Motif 13 (ADAMTS13). Among downregulated proteins, coagulation-related factors were prominent: Protein Z (PROZ, ~36% of control), Protein C (PROC, ~29%), Leukocyte Elastase Inhibitor (SERPINB1, ~43%), and Apolipoprotein C-IV (APOC4, ~59% of control). GO analysis revealed the same pattern, confirming that ECM remodeling was the primary increased biological process (NES ≈ +2.6, adj. *p*-value ≈ 2.4 × 10^−20^), alongside external encapsulating structure and ECM structural constituent (NES ≈ +3.05, adj. *p*-value ≈ 3.3 × 10^−20^), and collagen-containing ECM components were similarly enriched (NES ≈ +2.5, adj. *p*-value ≈ 6.7 × 10^−17^; Figure 2b). Cytosolic components were significantly enriched among downregulated molecular signatures, suggesting reduced abundance of proteins involved in glycolysis, protein synthesis, signal transduction, proteasomal degradation, cytoskeletal regulation, intracellular transport, and metabolic pathways. Hallmark and KEGG pathway analyses further highlighted key systemic alterations. The Hallmark Epithelial–Mesenchymal Transition (EMT) pathway, incorporating multiple ECM proteins, was strongly enriched (NES ≈ +2.39, adj. *p*-value ≈ 8.6 × 10^−9^). Other top increased pathways included fibrosis, angiogenesis, and complement/coagulation cascades (Figure 2c). Top downregulated pathways included signaling pathways related to cell proliferation/regeneration capacity, cytoskeletal function, endothelial function, and mechanical signal transduction (actin signaling: RAC, CDC42; PIK3/AKT/mTOR pathway).

### 2.3. Associations of the Serum Proteome with Time Elapsed Since Fontan Completion

**Figure 3:**
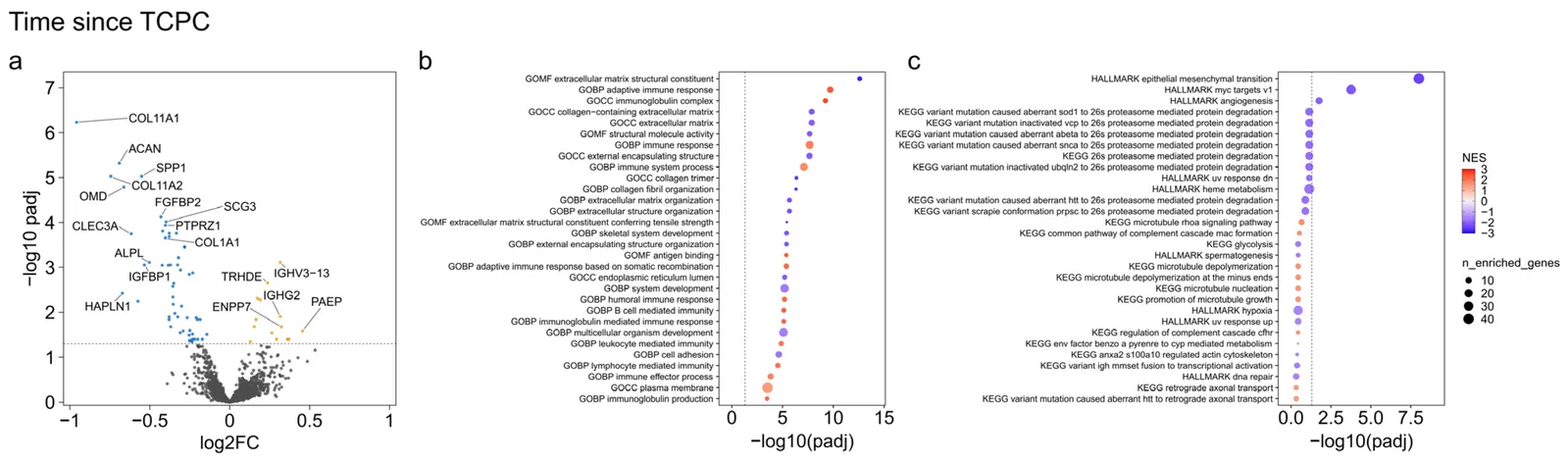
Associations of the proteome with time elapsed since Fontan completion. (**a**) Volcano plot displaying differential protein abundance in patients according to their Fontan duration (association with time since Fontan completion). Positive log_2_ fold change (log_2_FC) values indicate a positive association with the analyzed variable, whereas negative values indicate a negative association. The y-axis represents statistical significance (−log_10_ adjusted *p*-value), and the dashed horizontal line indicates the significance threshold (adjusted *p*-value = 0.05). (**b**) Gene set enrichment analysis (GSEA) of Gene Ontology (GO) categories. Red dots indicate positively enriched gene sets (normalized enrichment score, NES > 0), whereas blue dots indicate negatively enriched gene sets (NES < 0). The x-axis displays the statistical significance of enrichment (−log_10_ adjusted *p*-value). (GOBP = GO biological process, GOCC = GO cellular component, GOMF = GO molecular function). (**c**) GSEA of MSigDB (Molecular Signatures Database) Hallmark and KEGG (Kyoto Encyclopedia of Genes and Genomes) pathways. Red dots indicate positively enriched pathways (NES > 0), whereas blue dots indicate negatively enriched pathways (NES < 0). The x-axis displays the statistical significance of enrichment (−log_10_ adjusted *p*-value). For panels (**b**,**c**), dot size reflects the number of detected proteins contributing to the respective gene set or pathway, and n_enriched_genes denotes the subset of proteins contributing most strongly to the enrichment signal (leading edge proteins). Abbreviations according to UniProt [8].

Longer Fontan duration was associated with upregulation of proteins related to immune activation and systemic adaptation, including IGHV3-13, IGHG2, PAEP, ENPP7, and TRHDE. In contrast, longer Fontan duration was associated with downregulation of proteins related to ECM organization and tissue remodeling, such as COL1A1, COL11A1, COL11A2, ACAN, NCAN, HAPLN1, OMD, and SPP1, along with factors involved in growth factor signaling (IGFBP1, FGFBP2) and cell–matrix interaction (CLEC3A, PTPRZ1). The results are visualized in Figure 3, while detailed data are provided in Supplementary Tables S2–S4, Sheet S3. GO analysis (Figure 3b) confirmed that immune activation was the primary increased biological pattern in patients with a long Fontan duration (NES ≈ +2.41) and that among decreased molecular patterns were ECM-related pathways in those with a longer Fontan duration. Hallmark and KEGG pathway analyses supported those alterations from a functional perspective: Upregulated were pathways involved in the complement cascade (including the membrane attack complex (MAC)), as seen in chronic endothelial stress. The Hallmark EMT pathway was significantly decreased in those with a long time elapsed since TCPC vs. patients with a shorter time since TCPC. Downregulated in patients long after TCPC compared to those shortly after TCPC (thus, upregulated short after TCPC) were also pathways related to angiogenesis and hypoxia, heme metabolism, and glycolysis.

### 2.4. Associations of the Proteome with a Composite Risk Profile

**Figure 4:**
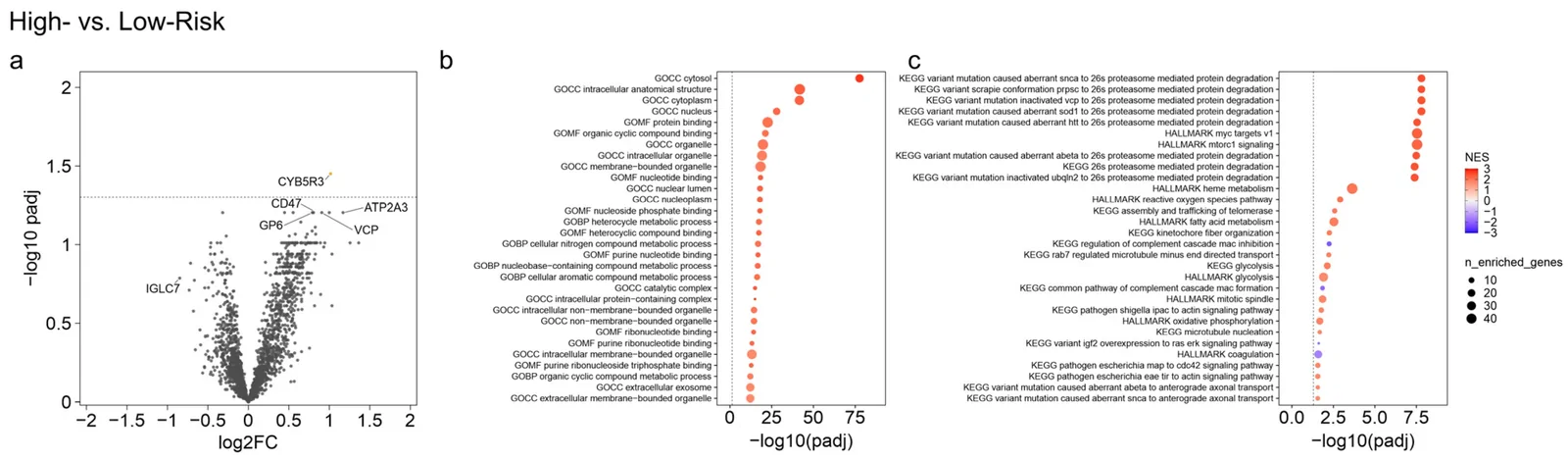
Associations of the serum proteome with the Fontan risk profile (high- vs. low-risk). (**a**) Volcano plot on the differential protein abundance in patients according to their risk score (association). Positive log_2_ fold change (log_2_FC) values indicate a positive association with the analyzed variable, whereas negative values indicate a negative association. The y-axis represents statistical significance (−log_10_ adjusted *p*-value), and the dashed horizontal line indicates the significance threshold (adjusted *p*-value = 0.05). (**b**) Gene set enrichment analysis (GSEA) of Gene Ontology (GO) categories. Red dots indicate positively enriched gene sets (normalized enrichment score, NES > 0), whereas blue dots indicate negatively enriched gene sets (NES < 0). The x-axis displays the statistical significance of enrichment (−log_10_ adjusted *p*-value). (GOBP = GO biological process, GOCC = GO cellular component, GOMF = GO molecular function). (**c**) GSEA of MSigDB (Molecular Signatures Database) Hallmark and KEGG (Kyoto Encyclopedia of Genes and Genomes) pathways. Red dots indicate positively enriched pathways (NES > 0), whereas blue dots indicate negatively enriched pathways (NES < 0). The x-axis displays the statistical significance of enrichment (−log_10_ adjusted *p*-value). For panels (**b**,**c**), dot size reflects the number of detected proteins contributing to the respective gene set or pathway, and n_enriched_genes denotes the subset of proteins contributing most strongly to the enrichment signal (leading edge proteins). Abbreviations according to UniProt [8].

As previously reported in our network-centric analysis of this cohort [6], and confirmed here using a complementary, hypothesis-free framework, Cytochrome b5 reductase 3 (CYB5R3) was the only individual protein to pass the multiple testing threshold when comparing individuals with a high- vs. low-risk profile defined by the composite risk score (log_2_FC ≈ +0.9–1.0, adj. *p*-value < 0.05, Figure 4). This effect persisted after the current preprocessing and covariate adjustment. No other single protein exceeded the FDR threshold in this contrast. Full statistics are provided in Supplementary Tables S2–S4 (Sheet S4). Functional enrichment in high-risk individuals (new to this analysis): To extend interpretation beyond single proteins, we also performed GSEA for the composite risk score analysis (which was not included in our prior work). In high-risk individuals, GSEA (GO) showed enrichment of analytes related to intracellular components (cytosol, nucleus, cytoplasm; NES ≈ +2.5–2.7, adj. *p*-value < 10^−27^) and metabolic/binding functions (protein/nucleotide binding, heterocycle metabolic processes; NES ≈ +2.3–2.5, adj. *p*-value < 10^−17^). KEGG pathway analysis highlighted involvement of upregulated proteins in functional pathways associated with response to oxidative stress (proteasome pathways) and energy metabolism and involvement of downregulated proteins in pathways associated with coagulation, complement, endothelial function, and proliferation (RAS/ERK signaling) in high-risk patients.

### 2.5. Associations of the Proteome with γ-Glutamyl Transferase (γGT) Levels

**Figure 5:**
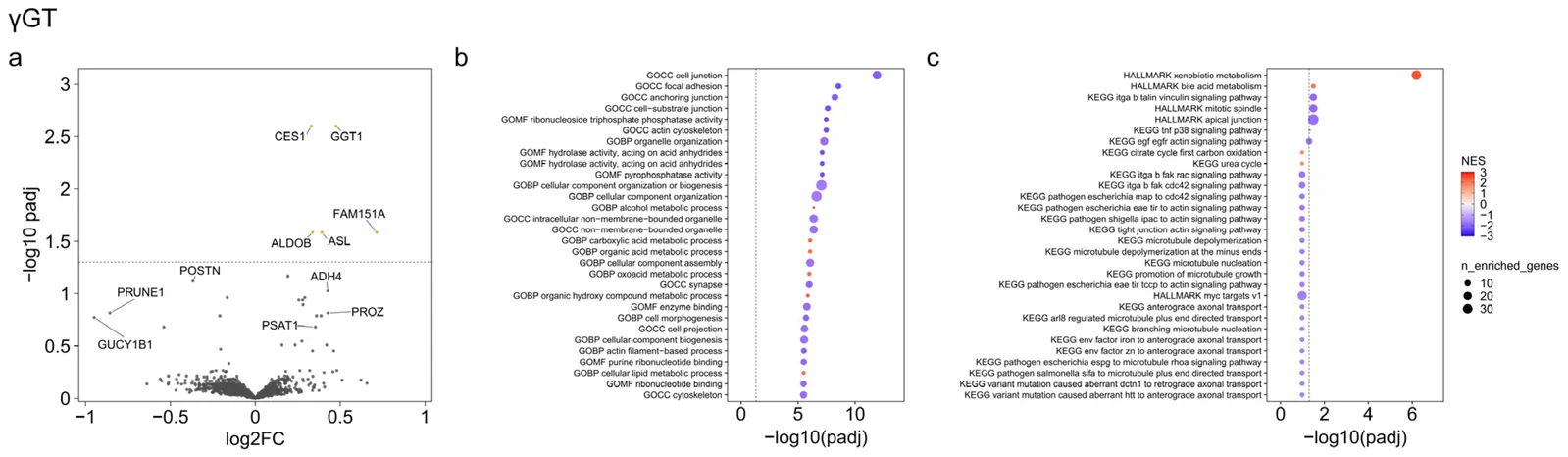
Associations of the serum proteome with γGT levels. (**a**) Volcano plot on the differential protein abundance in patients according to their serum γGT levels. Positive log_2_ fold change (log_2_FC) values indicate a positive association with the analyzed variable, whereas negative values indicate a negative association. The y-axis represents statistical significance (−log_10_ adjusted *p*-value), and the dashed horizontal line indicates the significance threshold (adjusted *p*-value = 0.05). (**b**) Gene set enrichment analysis (GSEA) of Gene Ontology (GO) categories. Red dots indicate positively enriched gene sets (normalized enrichment score, NES > 0), whereas blue dots indicate negatively enriched gene sets (NES < 0). The x-axis displays the statistical significance of enrichment (−log_10_ adjusted *p*-value). (GOBP = GO biological process, GOCC = GO cellular component, GOMF = GO molecular function). (**c**) GSEA of MSigDB (Molecular Signatures Database) Hallmark and KEGG (Kyoto Encyclopedia of Genes and Genomes) pathways. Red dots indicate positively enriched pathways (NES > 0), whereas blue dots indicate negatively enriched pathways (NES < 0). The x-axis displays the statistical significance of enrichment (−log_10_ adjusted *p*-value). For panels (**b**,**c**), dot size reflects the number of detected proteins contributing to the respective gene set or pathway, and n_enriched_genes denotes the subset of proteins contributing most strongly to the enrichment signal (leading edge proteins). Abbreviations according to UniProt [8].

There was a positive association of γ-glutamyl transferase (γGT) levels with levels of Carboxylesterase 1 (CES1, a functional marker of hepatic metabolic status), Aldolase B (ALDOB), and Peptidase M20 Domain Containing 1 (PM20D1), which is involved in protein turnover and signal modulation, when γGT was expressed continuously (Figure 5a). In the functional enrichment GO analysis, upregulated pathways were primarily associated with small-molecule and organic acid metabolic processes, including mitochondrial activity, energy metabolism, reactive oxygen species detoxification, and lipid metabolism, whereas downregulated pathways were related to ECM and cytoskeletal processes (Figure 5b). The Hallmark functional analysis suggested that patients with (moderately) higher γGT levels exhibited increased pathways involved in angiogenesis, complement activation, PI3K and RAS signaling and decreased pathways involved in epigenetic activation/gene response (Figure 5c).

### 2.6. ASA Therapy—Single Patient Findings

Patients with ASA intake (all four patients received ASA only and no vitamin K antagonist) showed upregulation of PROZ and downregulation of Eukaryotic Translation Initiation Factor 2 Subunit Alpha (EIF2S1) compared to those without ASA. No further proteins were differentially abundant. Due to subgroup size (single cases) this is only mentioned as brief information, and we refrain from visualization or further functional analyses.

### 2.7. Associations of the Proteome with Further Variables

Subgrouping the cohort according to ventricular morphology, ejection fraction, peak oxygen uptake, hemoglobin values, or platelet count did not show significant associations with proteomic analytes, neither did stratification with respect to intake of vitamin K antagonists (PROZ levels were non-significantly decreased, and EIF2S1 levels were non-significantly increased in those receiving vitamin K antagonists).

### 2.8. Brief Summary of the Findings

**Figure 6:**
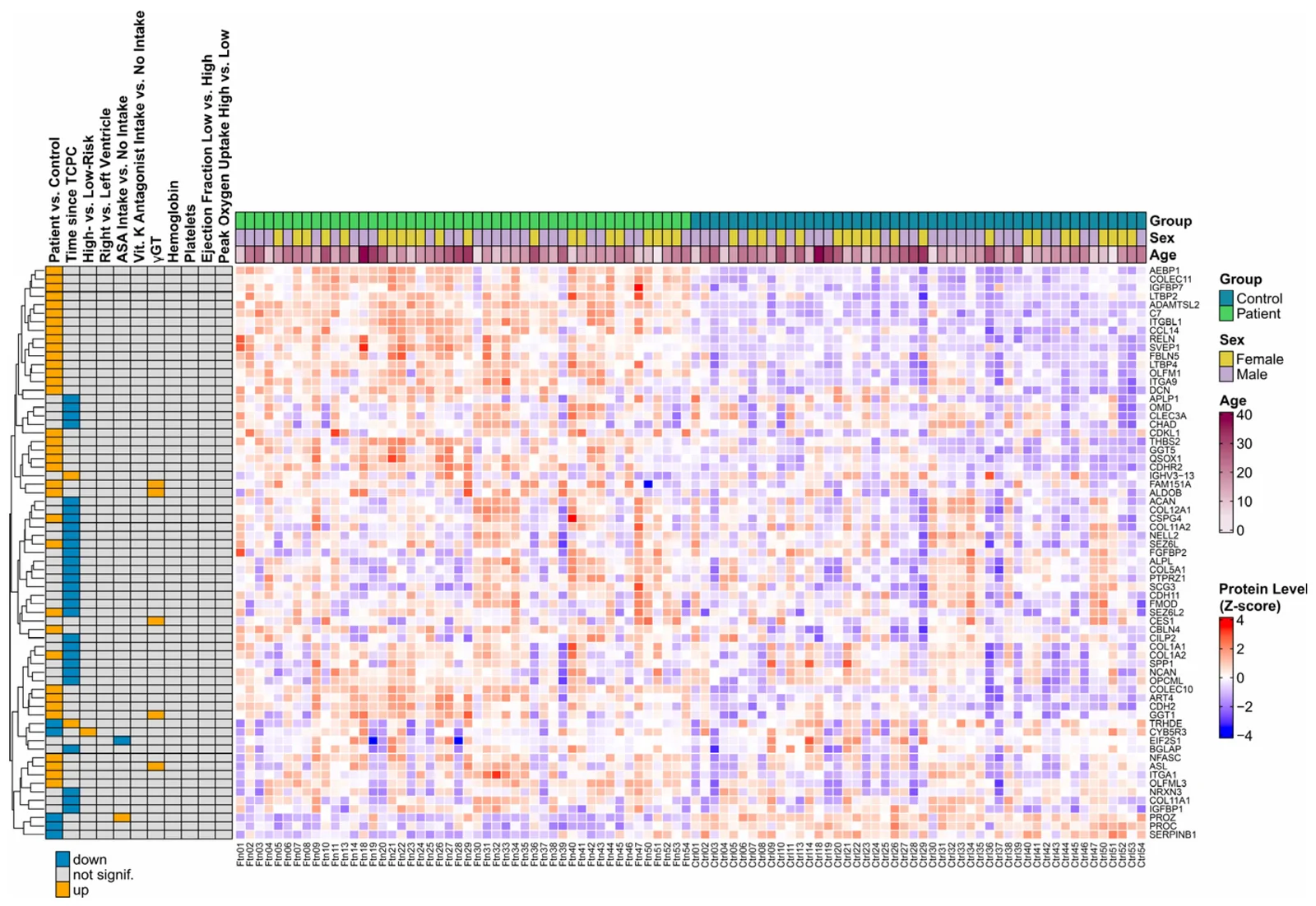
Per-sample heatmap of z-scaled expression levels for the 30 top differentially expressed proteins across all comparisons (selected by padj < 0.05). The left annotation indicates differential expression status for each contrast; the top annotation shows clinical variables. Proteins among the top 30 with padj < 0.05 which are increased in patients are marked in orange; those which are decreased in patients are marked in blue.

Collectively, the presented findings here suggest that the Fontan physiology is characterized by a persistent profibrotic baseline state, while high-risk phenotypes are associated predominantly with secondary immune and metabolic remodeling. Besides Fontan physiology, its duration and the composite risk score, there was just one variable (out of morphology, anticoagulants/antiplatelet therapy, exercise parameters, ejection fraction, and further laboratory values), namely γGT levels, associated with proteomic patterns. The heatmap (Figure 6) comprehensively visualizes our findings.

## 3. Discussion

This study complements and extends prior analyses of the circulating proteome in individuals with a Fontan circulation by applying a complementary, hypothesis-free framework [4] and by expanding subgroup phenotyping [6]. The key finding is that the Fontan circulation is associated with coordinated, multisystem proteomic remodeling, with stronger associations observed in individuals with longer time since Fontan completion and across clinical subgroups.

### 3.1. TGF-β-Driven ECM Pattern Inherent to Fontan

More than one third of all quantified proteins were differentially expressed between Fontan patients and controls, underscoring the magnitude of systemic biological remodeling in Fontan physiology. Using preranked GSEA with the measured proteome as the background, across the cohort, ECM organization and profibrotic signaling emerged as the dominant and most consistent signatures, supported by strong enrichment of ECM-related pathways and coordinated upregulation of fibrosis-associated proteins including ADAMTSL2, AEBP1, DCN, ITGBL1, and THBS2. Together with enrichment of EMT- and TGF-β-associated pathways, these findings support persistent activation of TGF-β-driven profibrotic remodeling programs in Fontan circulation, a pattern which is supported by previously described markers in Fontan, such as CD 44, Galectin 3, Matrix Metalloproteinase-7 or Matrix Metalloproteinase-8 [9,10,11].

The liver likely represents a central contributor to this TGF-β-driven biology. Recent single-cell transcriptomic analyses of Fontan-associated liver disease (FALD) demonstrated that hepatocytes exhibit early metabolic dysregulation and actively participate in TGF-β-superfamily signaling toward non-parenchymal cells, including hepatic stellate cells [12,13]. These observations support a model in which Fontan hemodynamics-associated altered hepatic metabolism promotes stellate cell activation and sustained ECM deposition through coordinated profibrotic signaling pathways. In parallel, our findings regarding APOC4 reduction and enrichment of lipid-associated pathways further support the concept that hepatic metabolic reprogramming is an integral component of Fontan pathophysiology rather than a secondary epiphenomenon. Of note, patients with γGT > 50 U/L in our cohort showed a trend towards a proteomic signature consistent with hepatocellular injury (mildly elevated ALT/γGT) and metabolic stress adaptation (elevated PM20D1). This is noteworthy, as transaminase elevations are generally mild in Fontan patients. Similarly, Ohuchi et al. reported that future onset of abnormal glucose metabolism was associated with both ALT/GPT and γGT in a multivariate model, despite only mild elevations of these enzymes [14]. These observations raise the question of whether even mild elevations of transaminases or γGT should be considered as clinically indicative of (mal)adaptive key biological processes in Fontan. In particular, persistently elevated γGT may indicate increased oxidative stress, reflecting its dual pro- and antioxidative role in glutathione metabolism. In FALD, sustained γGT elevation is associated with adverse outcomes and, when accompanied by declining platelet counts, may signal progressive fibrotic remodeling; conversely, γGT reduction under ursodeoxycholic acid therapy appears to mitigate fibrosis progression [15]. Beyond the liver, elevated γGT may reflect systemic oxidative stress: in congenital heart disease, including a few patients with single-ventricle lesions, mortality has been linked to higher γGT levels [16]. Importantly, while γ-GT aligned with the proteomic signal in our study, platelet count (as well as hemoglobin values) did not. Although preliminary, this may potentially indicate that γ-GT and its discussed role in pro- and antioxidative processes reflect aspects of Fontan physiology, particularly oxidative/hepatocellular stress, more readily than platelet-based surrogates of fibrosis (which are typically challenging to assess and interpret non-invasively in the Fontan circulation with elevated central venous pressure). Besides the hepatocytes, another system contributing to the TGF-β-driven biology may be the endothelium in general or the pulmonary vascular endothelium specifically. Proteins such as ADAMTS4, which are increased in pulmonary hypertension and also in pulmonary hypertension associated with congenital heart disease [17], may play a central role in TGF-β-driven mechanisms in these patients. Interestingly, our previously studied cohort of high-performing Fontan patients with a systemic left ventricle demonstrated a distinct molecular profile (as opposed to this study, which was an immune-based approach focusing on different molecules than the mass spectrometry-based approach presented here), characterized by mildly elevated soluble syndecan-1 in the absence of increased TGF-β signaling and without a dominant ECM remodeling signature [18]. Together with the observed negative correlation between soluble syndecan-1 and TGF-β, and together with decreased levels of leukemia inhibitory factor and nerve growth factor-β, this may indicate a more adaptive endothelial glycocalyx remodeling state and a low pro-angiogenic environment in clinically stable Fontan physiology and thus a fine-tuned TGF-β-mediated regulation of angiogenic and fibrotic pathways in those patients. In contrast, the present heterogeneous cohort, including patients with mixed ventricular morphology and greater clinical burden, revealed a systemic proteomic profile related to ECM and profibrotic pathway activation. One possible interpretation is that mild endothelial glycocalyx turnover may initially represent a compensatory response maintaining endothelial–matrix homeostasis and limiting excessive profibrotic signaling, whereas progression toward maladaptive Fontan physiology is associated with loss of this balance and transition toward persistent TGF-β-associated ECM remodeling. A link to the endothelial system is supported by the fact that, beyond ECM remodeling, the data presented here demonstrate converging alterations in endothelial, hemostatic, and immune pathways. Downregulation of anticoagulant and platelet-associated proteins including PROC, PROZ, RAP1B, and PLEK, together with enrichment of complement-associated proteins such as C7, COLEC10, and COLEC11, suggests persistent endothelial stress and dysregulated vascular homeostasis.

While reduced PROZ levels are likely influenced in part by long-term vitamin K antagonist therapy, which suppresses γ-carboxylation of several coagulation factors independently of hepatic synthetic capacity, an association between PROZ levels and lipid oxidation, previously described in Fontan patients, is also possible [19,20], potentially suggesting a contribution of membrane lipid oxidation to the broader pattern of chronic endothelial perturbation characteristic of the Fontan circulation [21]. To evaluate this hypothesis, lipidomic examinations would be required. So far, PROC and PROZ should be interpreted primarily as markers of pharmacologic vitamin K antagonist exposure rather than as evidence of disease-specific mechanisms. The same holds for the findings with respect to ASA intake: among the four patients receiving ASA (all without vitamin K antagonist), PROZ levels were higher, and EIF2S1 levels were lower than in patients not receiving ASA (the latter group consisting of patients receiving vitamin K antagonists). Given the very small subgroup sizes, these are descriptive observations only. No statistical significance after multiple testing correction is claimed. Given the near-universal use of vitamin K antagonists or antiplatelet therapy in individuals with a Fontan circulation, mechanistic conclusions are not drawn from these findings. In our prior study we presented one single patient who (due to non-compliance) was neither on anticoagulant nor on antiplatelet therapy. This person showed an isolated increase in levels of Elastin Microfibril Interfacer 1 (EMILIN1) [6]. Whether any of those proteins mentioned here is a candidate worth focusing on in future work, e.g., with respect to propensity to develop extracardiac conduit calcification [22] or liver disease, remains open at this point.

We observed higher ADAMTS13 levels on average. As a key regulator of von Willebrand factor (vWF) multimer size and platelet adhesion, ADAMTS13 occupies a central position within the vWF–ADAMTS13 axis, which has been implicated in the complex interplay between thrombosis, endothelial dysfunction, and bleeding in the Fontan circulation. Previous studies have demonstrated elevated vWF levels together with a reduction in high-molecular-weight multimers, a phenomenon exacerbated by (albeit mild) cholestasis and attenuated in patients receiving ASA therapy [23]. Although a direct effect of ASA on ADAMTS13 activity has not been demonstrated, and although our data are not validated and depend on small patient numbers, these findings may potentially support future work focusing on the hypothetical concept that endothelial dysfunction, liver disease, and dysregulation of the vWF axis are closely interconnected in Fontan pathophysiology.

In addition, enrichment of lectin complement pathway components and immune-related pathways supports the concept that innate and adaptive immune activation are integral components of Fontan biology rather than merely secondary inflammatory bystanders. This interpretation is further supported by recent liver single-cell transcriptomic studies demonstrating lymphocytic infiltration and activation of T- and NK-cell populations in FALD [24].

### 3.2. Associations of the Proteome with Time Elapsed Since Fontan Operation

In patients early after Fontan completion, the proteomic profile was dominated by ECM remodeling, angiogenic signaling, and growth factor pathways, consistent with active structural adaptation to chronically altered hemodynamics and non-pulsatile pulmonary blood flow. In contrast, long-term Fontan patients showed patterns related more to chronic immune activation and systemic metabolic adaptation, including increased immunoglobulin-related proteins and pathways linked to complement activation and cellular metabolism. The role of thymectomy in these patients may be an interesting point to examine.

The observed enrichment of Ectonucleotide pyrophosphatase/phosphodiesterase 7 (ENPP7) and lipid-associated pathways further suggests involvement of the gut–liver axis and intestinal metabolic adaptation in advanced Fontan physiology [25,26,27]. Chronic venous congestion, altered mesenteric flow and lymphatic dysfunction may contribute to this transition through intestinal congestion, altered mucosal permeability, and sustained low-grade immune activation, thereby linking cardiovascular, hepatic, intestinal, and lymphatic pathology into a unified multisystem disease process.

### 3.3. Associations of the Proteomic Pattern with Clinical Phenotyping

Individuals with a clinically unfavorable Fontan phenotype revealed associations of the serum proteome with an additional layer of biological remodeling. Rather than displaying a simple amplification of pathways related to ECM signatures, pathway-level analysis in high-risk patients indicated enrichment of analytes/patterns related to intracellular metabolic and redox-related pathways, including oxidative phosphorylation, glycolysis, fatty acid metabolism, and proteostasis-associated processes. We briefly restate the CYB5R3 finding here (previously reported in our network-centric analysis and reproduced under the present hypothesis-free framework) because it provides a mechanistically interpretable single protein anchor within these redox/metabolic pathways. CYB5R3, the strongest discriminator between clinical risk groups, is a key redox enzyme involved in lipid desaturation and mitochondrial oxidative metabolism and may reflect maladaptive responses to chronic oxidative stress and altered substrate utilization [6]. Together, these observations suggest that metabolic dysregulation represents a superimposed layer on a common fibrotic/endothelial baseline in Fontan. Such metabolic remodeling may contribute to progressive organ dysfunction and adverse clinical trajectories in advanced Fontan physiology. Remarkably, the composite clinical risk score rather than any single constituent (ventricular morphology, ejection fraction, peak exercise capacity, or laboratory indicators of relevant chronic desaturation or advanced (fibrotic) liver disease) stood out as a proteomic discriminator. This supports the view that risk in the Fontan circulation is multidimensional and that aggregated clinical information captures molecular variance more effectively than isolated metrics. Given potential contributions of local hemodynamics (e.g., wall shear stress) to endothelial activation and TGF-β processing, this suggests that the Fontan system reflects an interplay of structural, circulatory, and metabolic determinants culminating in distinct molecular phenotypes. Future work relating proteomic patterns to quantitative measures such as ventricular hypertrophy, myocardial strain, and 4D-flow-derived metrics may help disentangle these contributions [28,29].

Taken together, our findings delineate a system-level profile of the Fontan circulation. Cohort-wide signatures of ECM remodeling and endothelial dysregulation appear broadly shared, largely independent of ventricular morphology or clinical status. Associations with longer Fontan duration are characterized by more pronounced immune activation and systemic metabolic alterations, and clinically unfavorable phenotypes show relative enrichment of intracellular metabolic and redox-related perturbations rather than extracellular expansion alone. These are cross-sectional associations consistent with a model in which fibrosis/ECM remodeling represents a persistent substrate and immune-metabolic alterations become more prominent with longer duration. To establish temporal sequence or causality, longitudinal studies are required.

### 3.4. Limitations and Future Directions

This study has several limitations. First, the sample size is modest, an inherent limitation in rare diseases such as the Fontan population, and the cohort is intentionally heterogeneous (to allow comparison with a deliberately selected high-performing Fontan group [18]), which enhances generalizability but reduces power and introduces biological variability that cannot be fully modeled. Second, it is a secondary analysis of a previously published cohort using a complementary, hypothesis-free framework and should be viewed as extending (not independently replicating) prior findings. Third, patients and controls were recruited at different centers. Despite harmonized criteria, standardized pre-analytics, identical processing (at the same laboratory and at the same time), and blinding, the site and group are collinear, and unmeasured site-related confounding cannot be excluded. Fourth, we adjusted for MS batch and performed sensitivity checks, yet residual batch effects remain possible. Independent metabolomics on the same samples (unpublished data) showed patient/control separation with limited batch structure, which reduces (but does not eliminate) concern that batch alone explains our results. Fifth, the cross-sectional design precludes conclusions on causal or temporal inference. Associations with time elapsed since Fontan completion reflect duration-linked differences and not necessarily actual longitudinal progression. Sixth, technical constraints of data-dependent proteomics limit detection of very low-abundance cytokines/hormones and favor abundant proteins, potentially accentuating ECM signals. Enrichment analyses also depend on database choice and background definitions. We therefore used a preranked GSEA on the measured proteome only. Gene sets were intersected with the quantified proteins, and only those members contributed to the enrichment score. Thus, the effective “universe” is the measured proteome, not the genome-wide catalog. Still, enrichment analyses, while informative, rely on previously published disease models and may not fully capture mechanisms in poorly understood rare diseases like the Fontan circulation. Seventh, residual confounding is possible due to imperfect measurement of body composition and unmeasured factors (e.g., physical activity, edema). This may particularly hold for the Fontan physiology itself, which may alter plasma volume and hepatic function. In the hypothesized setting of (at least subtle) protein loss and particularly in the setting of chronic hypoxia and a respectively higher hematocrit, albumin/hematocrit adjustments are not entirely helpful. Matching patients and controls for body mass index and body composition would be an interesting approach. Eighth, we imputed the remaining missing values and based all inference on the imputed dataset. We did not perform dedicated sensitivity analyses without imputation or after excluding proteins with high imputation fractions. Therefore, while imputation enabled the inclusion of a broader set of proteins, we recognize that findings for proteins with substantial imputation warrant cautious interpretation and targeted validation. Ninth, circulating proteins cannot be attributed to specific organs, and medications, particularly long-term anticoagulation, may influence protein levels independently of disease biology. Integration of existing tissue-specific datasets, ideally across institutions, may help overcome the limitations associated with invasiveness and small sample size. Tenth, this is a discovery study without orthogonal laboratory validation of individual proteins. The biological plausibility of our findings is supported by related observations in an earlier, independent cohort of individuals with a Fontan circulation [30], which pointed to alterations in amino acid-linked pathways relevant to oxidative stress (e.g., methionine and transsulfuration intermediates) and ECM remodeling (e.g., proline/hydroxyproline and related metabolites, or taurine as an amino-sulfonic acid). However, it does not substitute for protein-level validation of the specific candidates identified here. Such validation will ideally be carried out in follow-up studies in independent (ideally larger, multicenter) cohorts, which also allow for larger subgroups (e.g., with respect to anticoagulation or antithrombotic therapy). Lastly, subgroup analyses are exploratory, and phenotyping or stratification according to a composite risk score remains critical to distinguish adaptive from maladaptive changes. Although within-comparison FDR was controlled, no global correction across all subgroup tests was applied, warranting validation in larger, prospective, multicenter cohorts. Longitudinal designs and integration with broader metabolomic and transcriptomic data will be important to test staged models and refine mechanistic interpretation.

## 4. Materials and Methods

### 4.1. Study Design and Population

This study was conducted and reported in accordance with the STROBE guidelines [31]. The underlying study cohort has been described previously [6]. In brief, this was a prospective, multicenter, cross-sectional observational study enrolling patients with a Fontan circulation and age- and sex-matched healthy controls. The present work represents an independent secondary analysis of this previously established cohort and specifically aimed to characterize systemic proteomic alterations associated with Fontan physiology, Fontan duration, and clinically unfavorable phenotypes using a complementary, hypothesis-free proteomic approach. A total of 48 Fontan patients were included, encompassing a spectrum of surgical modifications and clinical phenotypes, including individuals with prior Fontan conversion or aortic interventions. The patients were recruited at the Heart and Diabetes Center North Rhine-Westphalia, Bad Oeynhausen, Germany. Individually age- and sex-matched healthy volunteers with normal biventricular cardiac anatomy were recruited at an independent tertiary center (Medical University of Innsbruck, Innsbruck, Austria) (thus site = group; the separation of recruitment sites reflects an institutional move of the principal investigator (MM) during the enrollment period). Eligibility criteria, pre-analytical procedures such as overnight fasting status, venipuncture, tube type, processing times/temperatures, aliquoting, storage conditions, and data capture were harmonized a priori, and all samples were processed under identical standards of procedures, labeled and multiplexed in Tandem Mass Tag (TMT) batches (see Section 4.3) and analyzed on the same LC–MS platform with randomized channel assignment (operators blinded to group). Written informed consent was obtained from all participants prior to their inclusion in the study. Due to the exploratory nature of this proteomic investigation, no formal sample size calculation was performed.

### 4.2. Clinical Phenotyping

All participants underwent a standardized study visit including medical history, physical examination, and routine laboratory testing. In Fontan patients, additional investigations included transthoracic echocardiography, cardiac magnetic resonance imaging, cardiopulmonary exercise testing, and liver ultrasound (Table 1). Clinical risk stratification was based on a previously described composite Fontan risk score integrating 12 hemodynamic, anatomical, and functional parameters [32]. Minor adaptations of cut-off values were applied to improve discrimination within the present cohort, as previously reported [6]. Patients were categorized into favorable (≤3 points) and unfavorable (>3 points) clinical profiles. In addition to the predefined risk stratification, extended subgroup phenotyping was performed for the present analysis, including stratification by time elapsed since Fontan completion, ventricular morphology, ventricular function, peak oxygen uptake in treadmill exercise capacity testing, and selected laboratory parameters suggestive of the extent of desaturation or liver involvement (hemoglobin, platelet count, γGT).

### 4.3. Sample Collection and Proteomic Analysis

Sample collection and proteomic measurements have been described previously [6]. In brief, venous blood samples were obtained after overnight fasting. Serum was separated within 20 min by centrifugation and stored at −80 °C until analysis. Standardized operating procedures were applied across centers to minimize pre-analytical variability. Untargeted serum proteomic profiling was performed using a TMT 16-plex workflow. High-abundance proteins were depleted, followed by tryptic digestion and isobaric labeling. Peptides were analyzed by nano-liquid chromatography coupled to high-resolution Orbitrap mass spectrometry (Orbitrap Eclipse, Thermo Fisher Scientific, Vienna, Austria). Protein identification was performed against the UniProt human database [8], and relative quantification was based on TMT reporter ion intensities.

### 4.4. Statistical and Bioinformatic Analysis

In contrast to the previously published network-based and pathway-filtered analysis of this dataset, the present study applies an independent, complementary analytical framework centered on global differential expression and unbiased pathway enrichment across the full proteome. This approach was specifically designed to capture coordinated biological programs without reliance on predefined protein–protein interaction networks or cluster-based feature selection. All analyses were performed in R (version 4.4.1; R Foundation for Statistical Computing, Vienna, Austria). Protein intensities were log_2_-transformed and normalized prior to analysis. Proteins missing in more than four out of six TMT batches were excluded. The remaining missing values were imputed using the R package msImpute (version 1.14.0) with the “v2” algorithm, resulting in 1070 proteins with at least one imputed value across all subgroups. The fraction of imputed samples is reported for each protein in Supplementary Table S2. Differential expression analysis was conducted using the R package limma (version 3.60.4) [33]. Linear models included batch as covariate (batch effects in the PCA (Figure 1)) and relevant variables for testing of specific contrasts. Moderated *t*-statistics were used for inference. *p*-values were adjusted for multiple testing using the Benjamini–Hochberg procedure within each contrast. No multiplicity correction was applied across the set of exploratory contrasts. Proteins with false discovery rate (FDR)-adjusted *p*-values (q < 0.05) were considered statistically significant [34]. Additionally, fold change thresholds were applied in selected subgroup analyses. To enable system-level interpretation, gene set enrichment analysis (GSEA) was performed in a preranked framework (R package clusterProfiler (version 4.12.6) [35] in combination with the fast GSEA (fgsea) algorithm) using the signed moderated t-statistic from limma as the ranking metric. Gene sets were obtained from the Molecular Signatures Database (MSigDB) [36] via the R package msigdbr (version 10.0.2), including Hallmark, KEGG, and GO collections (org.Hs.eg.db, version 3.19.1). To avoid genome-scale bias, the background (universe) was restricted to the measured proteome, defined as all proteins with quantifiable signal prior to subgroup-specific filtering. For each contrast, gene sets were intersected with this universe, and only intersecting members contributed to the enrichment score. Enrichment scores were calculated using the weighted Kolmogorov–Smirnov statistics as implemented in fgsea, and normalized enrichment scores (NES) with FDR-adjusted q-values were reported (FDR q < 0.05). Within each contrast, fgsea reported Benjamini–Hochberg-adjusted q-values across the gene sets tested. No across-contrast multiplicity correction was applied. Leading edge subsets for all significantly enriched genes (FDR q < 0.5) were extracted from the fgsea output and are provided in Supplementary Tables S3 and S4 (last column, ‘enriched genes’). Protein-to-gene mapping used HGNC gene symbols (org.Hs.eg.db, version 3.19.1). Where multiple proteins mapped to the same gene symbol, the entry with the largest absolute t-statistic was retained to avoid duplication. For over-representation tests (where applicable), we used the same measured proteome universe rather than a genome-wide background. Analyses were performed across multiple biological comparisons, including Fontan versus control, time elapsed since Fontan operation, clinical risk groups, and extended subphenotypes. Visualization included PCA, heatmaps, volcano plots, enrichment plots, and pathway-level summaries.

## 5. Conclusions

The Fontan circulation is characterized by coordinated, systemic proteomic remodeling that extends beyond a purely hemodynamic disorder. Patterns related to ECM remodeling and TGF-β-associated profibrotic signaling emerged as dominant, cohort-wide features, suggesting that fibrosis represents a fundamental biological substrate of Fontan physiology rather than a mere marker of advanced disease. In contrast, longer time elapsed since Fontan completion and clinically unfavorable phenotypes were less associated with further amplification of patterns related to fibrotic signaling than with those related to enhanced immune activation, endothelial stress, and intracellular metabolic alterations with increased oxidative stress. These cross-sectional associations are consistent with a staged systems-biology framework, in which persistent structural/profibrotic remodeling coexists with more prominent immune-metabolic changes in individuals with longer Fontan duration. However, they do not establish temporal sequence or causality. Molecular phenotyping may complement conventional clinical assessment and help prioritize hypotheses for diagnosis, risk stratification, and therapeutic intervention, which warrant larger, prospective, and longitudinal validation across centers.

## Figures and Tables

**Table 1:** Overview of participant characteristics and composite Fontan risk score parameters.

Items	Unit	N	Patients	Controls
Age, mean (SD)	years	48	18.4 (7.6)	18.5 (7.6)
Female sex, n (%)		48	21 (44)	21 (44)
Weight, mean (SD)	kg	48	52.7 (19)	58.3 (17.2)
Height, mean (SD)	cm	48	152.5 (29.4)	165.8 (17.7)
BMI, mean (SD)	kg/m^2^	48	21.3 (6.3)	20.62 (3.0)
* Right ventricle, n (%)		48	22 (46)	
* Aortic Valve Regurgitation, n (%)		39	8 (21)	
Time elapsed since TCPC, mean (SD)	years	48	14.9 (7.65)	
* Open fenestration, n (%)		39	7 (18)	
Ejection fraction, mean (SD)	%	48	50 (10)	
* Ejection fraction < 50%, n (%)		48	23 (48)	
Cardiac index, mean (SD)	L/min/m^2^	40	1.95 (0.83)	
* Cardiac index < 2.5 L/min/m^2^, n (%)		39	26 (66)	
Significant AVVR, n (%)		39	8 (21)	
Peak SO_2_, mean (SD)	%	46	93 (4)	
* Peak SO_2_ < 90%, n (%)		46	8 (17)	
Minimal SO_2_ under exercise, mean (SD)	%	27	87 (6)	
* Collateral flow on angiography, n (%)		27	12 (44)	
* Tunnel dilation on angiography, n (%)		48	8 (17)	
* Central venous pressure >15 mmHg, n (%)		28	0 (0)	
* Diaphragmatic paresis, n (%)		48	0 (0)	
$\dot{V}$O_2_AT, mean (SD)	mL/kg/min	24	24 (6)	
Peak $\dot{V}$O_2_, mean (SD)	mL/kg/min	25	28 (6.6)	
% of predicted peak $\dot{V}$O_2_ ≥ 80, n (%)		25	5 (20)	
% of predicted peak $\dot{V}$O_2_ ≥ 50, n (%)		25	21 (84)	
* Peak $\dot{V}$O_2_ < 45% of normal, n (%)		25	2 (8)	
* Protein losing enteropathy, n (%)		25	5 (20)	
Vitamin K antagonist, n (%)		48	43 (90)	
Acetylsalicylic acid, n (%)		48	4 (8)	
No antithrombotic therapy, n (%)		48	1(2)	
Elastography ≥ 22 kPa, n (%)		20	4 (20)	
METAVIR Score ≥ 4, n (%)		18	11 (61)	
AST, mean (SD)	U/L	28	37 (14)	
ALT, mean (SD)	U/L	29	30 (16)	
AP, mean (SD)	U/L	18	138 (92)	
γGT, mean (SD)	U/L	25	68 (50)	
γGT ≥ 50 [U/L], n (%)		25	14 (56)	
Hemoglobin, mean (SD)	g/dL	36	15 (2)	
Hemoglobin ≥ 15 g/dL, n (%)		36	17 (47)	
Platelets, mean (SD)	1/µL	36	225 (102)	
Platelets ≤ 150,000/µL, n (%)		33	13 (39)	
Total protein, mean (SD)	g/dL	37	6.9 (1)	
Albumin, mean (SD)	g/dL	31	4 (0.7)	
Risk score ≤ 3, n (%)		48	37 (77)	

ALT, alanine transaminase; AP, alkaline phosphatase; AST: aspartate aminotransferase; AT, anaerobic threshold; AVVR, atrioventricular valve regurgitation; BMI: body mass index; γGT: gamma-glutamyl transferase; METAVIR, meta-analysis of histological data in viral hepatitis; N, sample size; SD, standard deviation; SO_2_, (pulse oximetric) oxygen saturation; TCPC, total cavopulmonal connection; $\dot{V}$O_2_, oxygen uptake. *, parameters constituting the 12-parameter risk score. One single patient received neither anticoagulation nor antiplatelet therapy. Further medications included ACE inhibitors (n = 11), L-thyroxine (n = 9), diuretics (n = 7), beta-blockers (n = 6), sildenafil (n = 1), and propafenone (n = 1). Note that of the 48 Fontan patients, 43 were receiving vitamin K antagonist therapy, and 4 were receiving acetylsalicylic acid (ASA). No patient received both therapies concurrently. One single patient received neither anticoagulant nor antiplatelet therapy.

## Data Availability

The original contributions presented in this study are included in the Supplementary Materials (Supplementary Table S5).

## Supplementary Materials

The following supporting information can be downloaded at: https://www.mdpi.com/article/10.3390/ijms27167461/s1.

## Abbreviations

The following abbreviations are used in the manuscript:

ADAMTS13	ADAM Metallopeptidase with Thrombospondin Type 1 Motif 13
ADAMTSL2	ADAMTS-Like Protein 2
AEBP1	AE Binding Protein 1
APOC4	Apolipoprotein C-IV
ASA	Acetylsalicylic Acid
COLEC10	Collectin-10
COLEC11	Collectin-11
DCN	Decorin
ECM	Extracellular Matrix
EIF2S1	Eukaryotic Translation Initiation Factor 2 Subunit Alpha
EMILIN1	Elastin Microfibril Interfacer 1
EMT	Epithelial–Mesenchymal Transition
ENPP7	Ectonucleotide Pyrophosphatase/Phosphodiesterase Family Member 7
FALD	Fontan-Associated Liver Disease
FDR	False Discovery Rate
GO	Gene Ontology
GSEA	Gene Set Enrichment Analysis
ITGBL1	Integrin Beta-Like Protein 1
KEGG	Kyoto Encyclopedia of Genes and Genomes
MSigDB	Molecular Signatures Database
NES	Normalized Enrichment Score
PM20D1	Peptidase M20 Domain Containing 1
PROC	Protein C
PROZ	Protein Z
TGF-β	Transforming Growth Factor Beta
THBS2	Thrombospondin-2
TMT	Tandem Mass Tag
